# Editorial: Syncope: Today and Tomorrow

**DOI:** 10.3389/fcvm.2020.00141

**Published:** 2020-08-06

**Authors:** Richard Sutton, Fabrizio Ricci, Artur Fedorowski

**Affiliations:** ^1^Department of Cardiology, National Heart & Lung Institute, Hammersmith Hospital, Imperial College, London, United Kingdom; ^2^Departments of Clinical Sciences, Lund University and Department of Cardiology, Skåne University Hospital, Malmö, Sweden; ^3^Department of Neuroscience and Imaging, Institute Advanced Biomedical Technologies, University G. d'Annunzio, Chieti, Italy

**Keywords:** syncope genetics, syncope pathophysiology, syncope presentation, orthostatic hypotension, carotid sinus syndrome, pacing reflex syncope, syncope after pacing

This volume of Frontiers Cardiovascular Medicine has been generated by the Editors with very close cooperation of many of the leaders in the field of syncope. The aim was to compile a substantial group of papers presenting recent work and contemporary reviews in the field. We felt a need to address a new focus on the genetics of vasovagal syncope (VVS) and aspects of its pathophysiology; notably, the role of the neuroendocrine system in the evolution of the event, the relative timing of circulatory and neurological events; new insights on the vexed problem of reflex atrioventricular block which really does exist despite denial in the recent past and the timing and depth of cerebral hypoxia. Further, we wanted to highlight aspects of the clinical presentation of syncope where developments have taken place. These are in the Emergency Department where a more collaborative approach reaps benefits; management of older patients presenting syncope requires particular care and thought and a thorough assessment of the interactions between syncope and head injury in the emergency presentation. Carotid sinus syndrome remains underappreciated and all too often ignored. Orthostatic hypotension also has new dimensions needing closer collaboration between neurologists and cardiologists as presently they seem to apply a different approach. In the last part, we felt that the difficult issues of how to pace and whom to pace is once again moving forward and the area of recurrent syncope in paced patients, another subject, unreasonably hitherto dismissed, required attention. Thanks to the contributors, we, the Editors, feel proud to have achieved our goal.

The volume begins with a section on the genetics of VVS in which Sheldon and Sandhu point to 3 candidate genes that are associated with VVS from a study of kindreds with high, multigenerational VVS prevalence.

A greater understanding of the pathophysiology of syncope follows with Benditt et al. in a wide-ranging review of neuroendocrine changes in vasovagal syncope. The most consistent literature finding, using tilt testing as a provocation, is a steep and early rise in epinephrine early in vasovagal reflex evolution. These authors raise the important question, yet to be answered, of whether this rise is a trigger or a response to evolving hemodynamics. It appears that this may only be revealed by much more frequent hormonal measurements during the induced attack.

van Dijk et al. show that in cardiac arrhythmias blood pressure falls more steeply than in VVS with syncope occurring in 8 s from the last systole in arrhythmias but may approach double this time in VVS from, in this case, onset of blood pressure fall.

Sutton addresses the reality and unexpectedly high frequency of reflex atrioventricular block (RAVB) in syncope, where 20% of older candidates for pacemaker therapy of VVS show this arrhythmia. Features of RAVB are described in order to aid diagnosis.

The section closes with a review of non-invasive cerebral oximetry assessment in syncope and orthostatic intolerance syndromes, with tilt as provocation, emphasizing that there are important reductions in cerebral oxygenation before the onset of hemodynamic disturbance in VVS and in Postural orthostatic tachycardia without blood pressure fall (Kharraziha et al.).

The following section offers studies on clinical presentation of syncope including that in the Emergency Department from Canada in which Sandhu and Sheldon give great importance to cooperation between disciplines to achieve the best possible care for the emergency presentation of syncope and report that, within 30 days, ~0.8% of patients die and 10.3% suffer a serious adverse event.

McCarthy et al. from Dublin, Eire report on nearly 5,000 older patients with syncope who were recruited into the Irish Longitudinal study of Aging. They found syncope to be common in the older patient, that its occurrence has an adverse effect of quality of life, most marked with two recent episodes, which is, in turn, influenced by fear of falling, a facet open to specific therapy.

A study of the problem of coincidence of syncope and head injury from Poland concludes that the short-term prognosis of syncope with head injury is similar to that of head injury alone but the long-term prognosis in these two conditions separates with syncope leading to head injury being substantially worse than head injury without syncope. In multivariable Cox regression analysis, syncope was one of the strongest independent predictors of long-term mortality (Furtan et al.).

The subsequent section is on Orthostatic hypotension (OH); drawing from the now very large series of patients in Malmö, Sweden who have been investigated for syncope and orthostatic intolerance. Torabi et al. separated classical OH from delayed OH finding surprisingly one-quarter of the whole group of patients present orthostatic hypotension of the two types. However, classical OH patients are older, more often have supine hypertension, pathologic Valsalva maneuver, Parkinson's disease, pacemaker-treated arrhythmia, and lower glomerular filtration rate. Classical OH is associated with increased vasopressin and epinephrine during head-up tilt, but blunted increase in norepinephrine. Their findings add to the distinction between the two clinical syndromes.

The second part of the section is on Carotid sinus syndrome which deserves much more attention than it receives. Parry, in his paper, elegantly recounts the history of this syndrome and considers whether pacing is ever required. This is a question not asked by Guidelines. Common misunderstandings are exposed. Carotid sinus hypersensitivity and carotid sinus syndrome are separated, implying different management approaches. Many old concepts are challenged, and the final recommendation is that pacing is probably indicated in some patients.

The final section on therapy provides reflections by Barón-Esquivias et al. on pacing for vasovagal syncope in the light of the SPAIN trial. They remain confident in the positive results of the SPAIN trial which showed pacing benefit in a randomized controlled methodology for younger patients than were included in previous trials without selection by insertable ECG loop recorder. The pacing system used a sensing system that detects right ventricular volume; a smaller volume triggers pacing at higher than the prevailing rate. A previous acute study has shown that this sensor permits earlier intervention by pacing than waiting for bradycardia which has been well-demonstrated to occur late in the evolution of VVS. The sensing system is known as closed loop (CLS) by Biotronik, Berlin, Germany. The current practice in Seville, Spain is to offer this type of dual-chamber pacemaker to 20% of their highly symptomatic referred patients.

The other study in this section raises another ignored problem that of recurrent syncope in patients who have been paced. The Malmö, Sweden group examine this in their large series of syncope and orthostatic intolerance patients. Thirty-nine patients (2.3% of the whole group, aged 65.6 years, 39% female) presented syncope recurrence after pacing from a database of 1,705 patients. In assessment of the cause none had pacemaker failure of any type; in 36 the cause was found by careful examination using cardiovascular autonomic testing which included Valsalva maneuver, active standing, carotid sinus massage, and tilt-testing. OH was the most common cause of recurrent syncope in 16 (41%) with VVS confirmed in 12 (31%). Three patients were not diagnosed. The authors advise a complete work-up such as this described in all paced patients with recurrent syncope (Yasa et al.).

We, the Editors, earnestly hope that, here, we have provided compelling reading for those interested in syncope.

## Author Contributions

All authors listed have made a substantial, direct and intellectual contribution to the work, and approved it for publication.

## Conflict of Interest

The authors declare that the research was conducted in the absence of any commercial or financial relationships that could be construed as a potential conflict of interest.

